# Co-production of hydrogen and ethanol by *pfkA*-deficient *Escherichia coli* with activated pentose-phosphate pathway: reduction of pyruvate accumulation

**DOI:** 10.1186/s13068-016-0510-5

**Published:** 2016-04-29

**Authors:** Balaji Sundara Sekar, Eunhee Seol, Subramanian Mohan Raj, Sunghoon Park

**Affiliations:** Department of Chemical and Biomolecular Engineering, Pusan National University, 2, Busandaehak-ro 63 beon-gil, Geumjeong-gu, Busan, 46241 Republic of Korea; Centre for Research and Development, PRIST University, Trichy–Thanjavur Road, Vallam, Thanjavur, TN 613 403 India

**Keywords:** Biohydrogen, Co-production of hydrogen and ethanol, Glycolysis, Pentose-phosphate pathway, NADPH production, *Escherichia coli*

## Abstract

**Background:**

Fermentative hydrogen (H_2_) production suffers from low carbon-to-H_2_ yield, to which problem, co-production of ethanol and H_2_ has been proposed as a solution. For improved co-production of H_2_ and ethanol, we developed *Escherichia coli* BW25113 Δ*hycA* Δ*hyaAB* Δ*hybBC* Δ*ldhA* Δ*frdAB* Δ*pta*-*ackA* Δ*pfkA* (SH8*) and overexpressed Zwf and Gnd, the key enzymes in the pentose-phosphate (PP) pathway (SH8*_ZG). However, the amount of accumulated pyruvate, which was significant (typically 0.20 mol mol^−1^ glucose), reduced the co-production yield.

**Results:**

In this study, as a means of reducing pyruvate accumulation and improving co-production of H_2_ and ethanol, we developed and studied *E. coli* SH9*_ZG with functional acetate production pathway for conversion of acetyl-CoA to acetate (*pta*-*ackA*^+^). Our results indicated that the presence of the acetate pathway completely eliminated pyruvate accumulation and substantially improved the co-production of H_2_ and ethanol, enabling yields of 1.88 and 1.40 mol, respectively, from 1 mol glucose. These yields, significantly, are close to the theoretical maximums of 1.67 mol H_2_ and 1.67 mol ethanol. To better understand the glycolytic flux distribution, glycolytic flux prediction and RT-PCR analyses were performed.

**Conclusion:**

The presence of the acetate pathway along with activation of the PP pathway eliminated pyruvate accumulation, thereby significantly improving co-production of H_2_ and ethanol. Our strategy is applicable to anaerobic production of biofuels and biochemicals, both of which processes demand high NAD(P)H.

**Electronic supplementary material:**

The online version of this article (doi:10.1186/s13068-016-0510-5) contains supplementary material, which is available to authorized users.

## Background

Biological H_2_ production can be accomplished via dark fermentation, photo-fermentation, or biophotolysis. Among these approaches, dark fermentation is considered the most promising, due to its simple bioreactor configuration and operation and, above all, fast H_2_ production rate [1–3]. Its commercial application, however, has a critical drawback: the low glucose-to-H_2_ production yield [4]. The theoretical maximum with facultative anaerobes such as *Enterobacter* sp. is 2 mol H_2_ mol^−1^ glucose, and that with strict anaerobes such as *Clostridia* sp. is 4 mol H_2_ mol^−1^ glucose [5]. Energy recovery is <40 % even with 4 mol H_2_ mol^−1^ glucose, and this makes H_2_ production less attractive compared to the production of other biofuels such as ethanol and butanol [6, 7]. To address the low H_2_ production yield from glucose, introduction of heterologous pathways such as ferredoxin- or NAD(P)H-dependent H_2_ production in *E. coli* has been attempted [8, 9]. Despite functional in *E. coli*, the heterologous pathways were highly inefficient and no practical improvement in H_2_ yield was achieved. From process development aspect, hybrid systems such as dark- plus photo-fermentation, hythane process (H_2_ in the first stage and methane in the second), among others, have been suggested; with most of these hybrid systems unfortunately, scale-up is problematic, due to the requirement of complex instrumentation and/or reactor operation [10–12]. As an alternative solution to the introduction of heterologous pathways or hybrid process development, we have suggested co-production of H_2_ and ethanol in a simple, single-reactor system [13]. Similar approaches but with different carbon source or target products have been reported. For example, from glycerol which is a more reduced substrate than glucose, co-production of H_2_ and ethanol by *E. coli* [14] and *Klebsiella* sp. [15] has been studied. Equimolar production of H_2_ and ethanol at 1 mol mol^−1^ glycerol was obtained successfully [14]. However, due to its limited supply, glycerol cannot be used for sustainable and renewable energy production. Co-production of H_2_ and acetaldehyde with glucose as carbon source has also been reported. However, in this case, acetaldehyde should be chemically reduced to ethanol to be used as fuel [16].

**Fig. 1:**
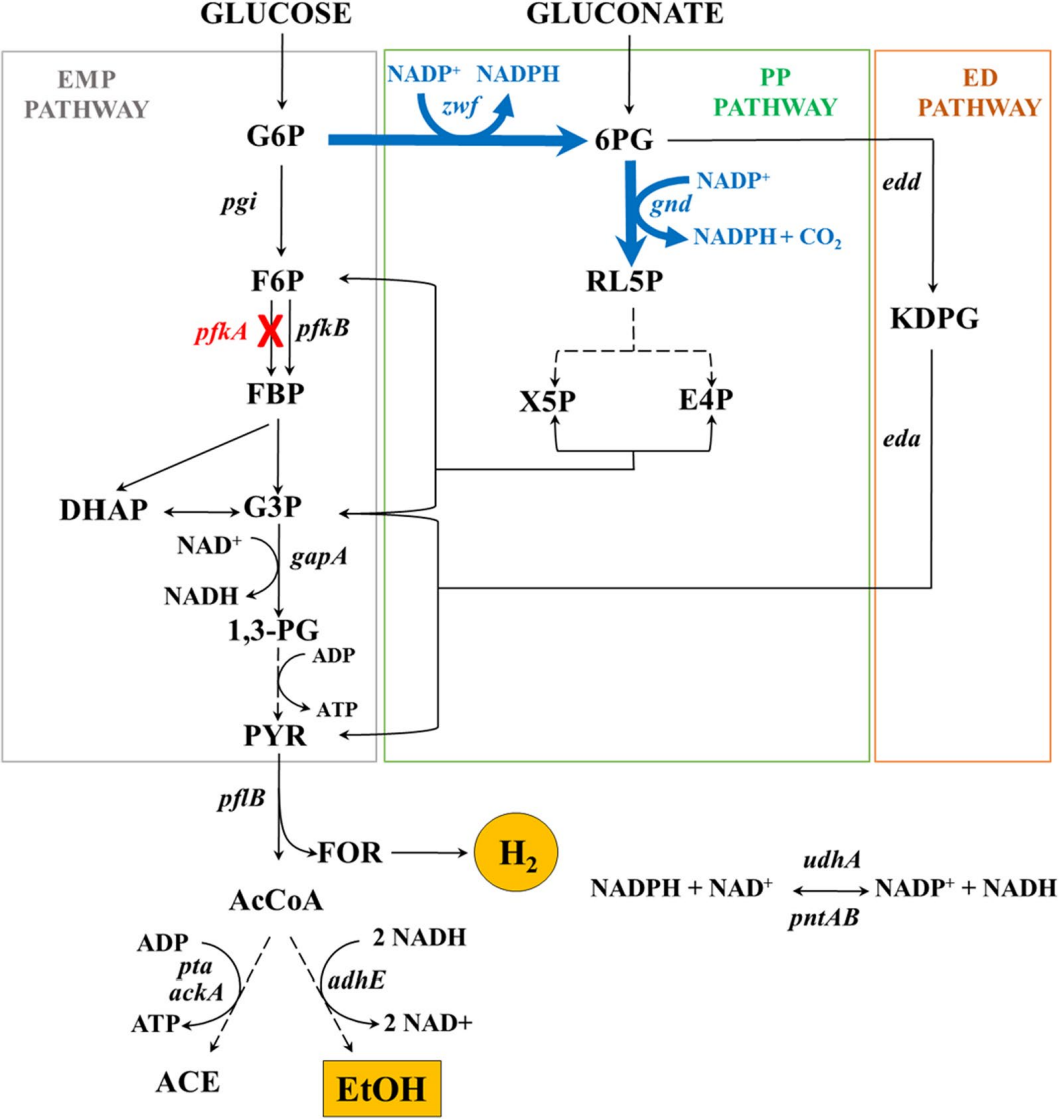
Strategy for promoting carbon flux through PP pathway to improve co-production of H_2_ and ethanol. EMP pathway was down-regulated by *pfkA* deletion (*red*) and PP pathway was activated by overexpression of *zwf* and *gnd* (*blue*). Genes: *pgi*—phosphoglucose isomerase, *pfk*—phosphofructokinase, *gapA*—glyceraldehyde-3-phosphate dehydrogenase, *pta*—phosphotransacetylase, *ackA*—acetate kinase, *adhE*—alcohol dehydrogenase, *zwf*—glucose-6-phosphate dehydrogenase, *gnd*—6-phosphogluconate dehydrogenase, *edd*—6-phosphogluconate dehyratase, *eda*—Entner–Doudoroff aldolase, *udhA*—soluble transhydrogenase, *pntAB*—membrane-bound transhydrogenase. Metabolites: G6P—glucose-6-phosphate, F6P—fructose-6-phosphate, FBP—fructose-1,6-bisphosphate, DHAP—dihydroxyacetone phosphate, G3P—glyceraldehyde-3-phosphate, 1,3-PG—1,3-bisphosphoglycerate, PYR—pyruvate, FOR—formate, H_2_—hydrogen, AcCoA—acetyl CoA, ACE—acetate, EtOH—ethanol, 6PG—6-phosphogluconate, RL5P—ribulose-5-phosphate, X5P—xylose-5-phosphate, E4P—erythrose-4-phosphate, KDPG—2-Keto-3-deoxy-6-phosphogluconate

Under anaerobic conditions, most glucose is metabolized via the Embden–Meyerhof–Parnas (EMP) pathway in facultative *Enterobacter* sp. including *Escherichia coli*. In this pathway, 1 mol of glucose is converted to 2 mol of pyruvate, and 2 mol of NADH is generated. Under anaerobic condition, pyruvate is further metabolized to acetyl-CoA and formate, from which ethanol, acetate, and H_2_ are produced (Fig. 1). In theory, for redox neutrality, 1 mol of acetate, 1 mol of ethanol, and 2 mol of H_2_ can be produced from 1 mol of glucose. For production of 2 mol of ethanol (instead of 1 mol of ethanol plus 1 mol of acetate), more NAD(P)H (i.e., 4 mol) must be generated during glycolysis, which is possible when glucose is metabolized through the pentose-phosphate (PP) pathway. However, the theoretical maximum yield for co-production of H_2_ and ethanol, according to carbon and energy balance, is 1.67 mol mol^−1^ each, not 2.0 mol mol^−1^. It is because that, for production of more NAD(P)H in the oxidative PP pathway, some carbon needs to be sacrificed and converted to carbon dioxide (CO_2_) [17]. In a previous study, in order to completely block the EMP pathway, we attempted to disrupt *pgi*, but the strain could not grow under anaerobic conditions [13]. Therefore, we deleted the major phosphofructokinase isozyme, PfkA, in *E. coli* BW25113 Δ*hycA* Δ*hyaAB* Δ*hybBC* Δ*ldhA* Δ*frdAB* (designated as SH5), so as to divert carbon flux to the PP pathway. Further, we also eliminated the acetate production pathway (*pta*-*ackA*) and overexpressed Zwf and Gnd, two major enzymes in the PP pathway. The resulting recombinant mutant SH8*_ZG (*E. coli* BW25113 Δ*hycA* Δ*hyaAB* Δ*hybBC* Δ*ldhA* Δ*frdAB* Δ*pta*-*ackA* Δ*pfkA* overexpressing *zwf* and *gnd*) could successfully co-produce ethanol (1.38 mol mol^−1^) and H_2_ (1.32 mol mol^−1^) from glucose, without acetate. However, a substantial amount of pyruvate (0.18 mol mol^−1^) was always produced, thus significantly reducing the co-production yields of H_2_ and ethanol [18].

In the present study, as a means of eliminating pyruvate accumulation and improving co-production yields, we developed a new *E. coli* mutant (designated SH9) with an intact acetate production pathway (*E. coli* BW25113 Δ*hycA *Δ*hyaAB* Δ*hybBC* Δ*ldhA* Δ*frdAB* Δ*pfkA*) from SH5 and evolved the strain (SH9*) for growth under anaerobic conditions. After Zwf and/or Gnd was overexpressed in SH9*, the recombinant strain was investigated for co-production of H_2_ and ethanol under various induction conditions. The flux distributions among the three glycolytic pathways (EMP, PP, and ED) as well as transcription of the major enzymes in these pathways were also analyzed. Additionally, the effects of the disruption of the Entner–Doudoroff (ED) pathway on activation of the PP pathway and co-production of H_2_ and ethanol were evaluated.

## Results and discussion

### Adaptive evolution of SH9 strain for anaerobic growth

**Fig. 2:**
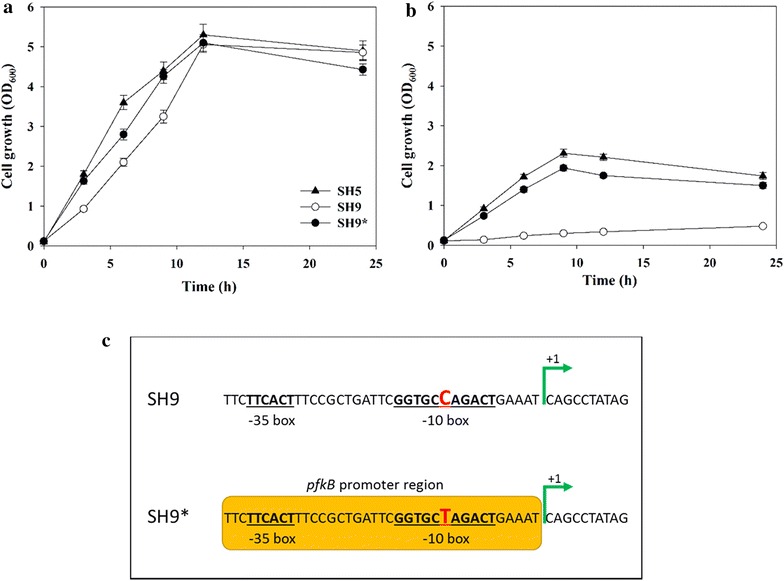
Growth of host strains (SH5, SH9, and SH9*) in glucose-containing M9 medium and mutation occurred in SH9*. Refer to Table 1 for the genotype of each strain. **a** Growth under aerobic condition, **b** growth under anaerobic condition and **c** difference in genome sequence between SH9 and SH9* (adapted SH9 strain). Adaptation introduced a single-point mutation only in the promoter region of *pfkB,* from ‘C’ to ‘T’. The mutated nucleotide is marked in *red*

SH9 was constructed by deleting PfkA, the major phosphofructokinase in the SH5 strain developed in a previous study [19] (Table 1). Under aerobic conditions, SH9 grew similarly to the parent strain (SH5) (Fig. 2a); however, under anaerobic conditions, it grew very slowly (Fig. 2b). In order to enhance anaerobic cell growth, SH9 was adapted to anaerobic conditions by transferring the culture to new media every 12–18 h. In the course of the adaptation, SH9 gradually recovered its growth, and, after ~15 transfers, it was able to grow at a rate similar to that of the SH5 strain (Fig. 2b). The adapted SH9 strain was designated SH9*.

**Table 1:** List of strains and plasmids used in this study

Strains and plasmids	Description	Source
SH5	*E. coli* BW25113 Δ*hycA* Δ*hyaAB* Δ*hybBC* Δ*ldhA* Δ*frdAB*	[31]
SH5_ZG	SH5/pEcZG	This study
SH9	SH5Δ*pfkA*	
SH9*	SH9—adapted for anaerobic growth	
SH9*_G	SH9*/pEcG	
SH9*_Z	SH9*/pEcZ	
SH9*_ZG	SH9*/pEcZG	
SH9*_ZGU	SH9*/pEcZGU	
SH10_ZG	SH9* Δ*eddeda*/pEcZG	
Plasmids		
pDK7	Bacterial expression plasmid, Cm resistant	[32]
pEcG	pDK7 carrying *gnd* of *E. coli* BW25113	[18]
pEcZ	pDK7 carrying *zwf* of *E. coli* BW25113	
pEcZG	pDK7 carrying *zwf,* and *gnd* of *E. coli* BW25113	
pEcZGU	pDK7 carrying *zwf,* *gnd* and *udhA* of *E. coli* BW25113	This study

The change in genotype during the adapted evolution was analyzed by sequencing of the SH9 and SH9* genomes. Surprisingly, only a single-nucleotide mutation in the promoter region of *pfkB* was identified (Fig. 2c): the nucleotide ‘C’ in the −10 box of the *pfkB* promoter in SH9 had been converted to ‘T’ in SH9*. PfkB, the minor isozyme of PfkA, is known to be expressed in the stationary growth phase [20]. It is believed that this promoter-region mutation increased the transcription of *pfkB*, thus allowing SH9* to metabolize glucose through the EMP pathway and support cell growth (Fig. 2c). A similar mutation has been reported in other *E. coli* strains lacking *pfkA* [18, 21].

### Operation of PP pathway by overexpression of Zwf and Gnd in SH9*

In SH9*, pyruvate accumulation was fully eliminated and H_2_ production, thereby, was significantly improved to 1.68 mol mol^−1^ (vs. 1.01 mol mol^−1^ by SH8*). However, nearly equimolar acetate and ethanol, 0.85 and 0.78 mol mol^−1^, respectively, were produced (Table 2). This result suggests that, in SH9*, NAD(P)H supply is not sufficient to push carbon flow to ethanol at the acetyl-CoA node. Although PfkA was deleted, the expression of PfkB had to have been upregulated substantially to a level sufficient to compensate for the absence of PfkA. To increase the carbon flux to the PP pathway and produce more NAD(P)H, Zwf and Gnd, the rate-limiting enzymes in the PP pathway, were overexpressed in SH9* (Fig. 1). SH9*_ZG, the recombinant strain overexpressing Zwf and Gnd in SH9*, showed greatly enhanced ethanol and H_2_ production: to 1.40 mol mol^−1^ ethanol from 0.85 mol mol^−1^, and to 1.88 mol mol^−1^ H_2_ from 1.68 mol mol^−1^, respectively (Table 2). At the same time, acetate production was greatly reduced, from 0.78 to 0.15 mol mol^−1^. The drastic reduction of acetate production in SH9*_ZG indicates that the enhanced NAD(P)H by operation of the PP pathway had been utilized for the conversion of acetyl-CoA to ethanol rather than to acetate. Yeast extract (1 g L^−1^) added to the culture medium could have contributed to the co-production yield, though its impact should have been marginal. The glucose concentration applied in the current experiment was 5 g L^−1^, while that of carbohydrate in 1 g of yeast extract was < 0.16 g only [22]. Energy recovery for the co-production of H_2_ and ethanol by SH9*_ZG corresponds to ~80 %; this is much higher than that of sole production of H_2_ (<40 %) at the theoretical maximum of 4 mol mol^−1^ glucose (by strict anaerobes such as *Clostridia* sp.) or comparable to that of ethanol production (~90 %) at the theoretical maximum of 2 mol mol^−1^ glucose (by ethanologenic *E. coli*, *Zymomonasmobilis* or *Saccharomyces cerevisiae*) [5, 23, 24].

**Table 2:** Comparison of metabolites yield of recombinant SH5, SH8*, and SH9* strains

Strain^b^	Overexpressed gene	Yield of metabolites^a,d^ (mol mol^−1^)			
		H_2_	Ethanol	Acetate	Pyruvate
SH5	–	1.44	0.79	0.67	–
	*zwf* and *gnd*	1.60	1.09	0.35	–
SH8*^c^	–	1.01 (0.59)	0.89 (0.48)	–	0.73 (1.36)
	*zwf*	1.20 (0.57)	1.18 (0.49)	–	0.41 (1.38)
	*gnd*	1.05 (0.99)	0.96 (0.79)	–	0.67 (1.07)
	*zwf* and *gnd*	1.32 (0.98)	1.38 (0.81)	–	0.18 (1.05)
SH9*	–	1.68 (1.68)	0.85 (0.51)	0.78 (1.37)	–
	*zwf*	1.76 (1.75)	0.80 (0.52)	0.87 (1.45)	–
	*gnd*	1.78 (1.64)	0.87 (0.68)	0.71 (1.13)	–
	*zwf* and *gnd*	1.88 (1.70)	1.40 (0.65)	0.15 (1.28)	–
SH10	*zwf* and *gnd*	1.57	1.30	0.38	–

^a^Yields of metabolites were calculated from three individual experiments and the standard deviation was less than 10 %

^b^Refer to Table 1 for the genotype of the strains

^c^Data for SH8* were obtained from the previous study and presented for comparison [18]

^d^Yields of metabolites in parentheses were obtained with gluconate as carbon source

Similar experiments with overexpression of Zwf or Gnd individually were also carried out (Table 2). With glucose as the carbon source, the performance of SH9*_Z or SH9*_G was not much different from that of SH9* (the host strain), indicating that expression of both Zwf and Gnd is required for diversion of carbon flux to the PP pathway. However, fermentation with gluconate, which bypasses Zwf and enters the PP pathway after its conversion to gluconate-6-phosphate, showed different ethanol and acetate production results according to the expression of Zwf and/or Gnd. This can be explained in two ways. First, compared with the case with glucose as the carbon source, much more acetate than ethanol was produced. Gluconate is more oxidized than glucose, generating less NAD(P)H during glycolysis. Less ethanol production with gluconate than with glucose confirms again that the production of ethanol relative to acetate is determined by the NAD(P)H supply in the glycolysis of the carbon substrate. Second, in SH9_G and SH9_ZG, where Gnd was overexpressed, ethanol production from gluconate increased while acetate production decreased. Both of these observations constitute strong evidence that the PP pathway and subsequent NAD(P)H supply are important in forcing the carbon flux to ethanol at the acetyl-CoA node.

It was also interesting to study the differential behaviors of SH8* and SH9* as a way to reveal the influence of the acetate pathway in the glycolytic flux distribution. As shown in Table 2, with SH8*, overexpression of Zwf, but not Gnd, increased ethanol production and reduced pyruvate production when glucose was used as the carbon source. With SH9*, on the other hand, overexpression of either Zwf or Gnd did not cause significant changes in metabolite production. When gluconate was used as the carbon source, Gnd overexpression in SH8* (SH8*_G and SH8*_ZG) increased ethanol production by reducing pyruvate accumulation. On the other hand, overexpression of Gnd in SH9* (SH9*_G and SH9*_ZG) resulted in only marginal changes in the production of ethanol and acetate. These observations suggest that the presence of an ATP-producing and acetate biosynthetic pathway influences the carbon distributions among the EMP, PP, and ED pathways and results in correspondingly varied ethanol production.

### Effect of differential expression of Zwf and Gnd on co-production of H_2_ and ethanol

**Fig. 3:**
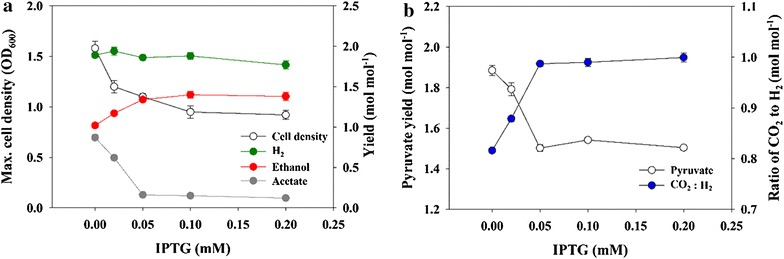
Metabolites yield of SH9*_ZG induced with varying concentrations of IPTG. **a** Final cell density, H_2_, ethanol and acetate. **b** Pyruvate (ethanol + acetate) yield and ratio of produced CO_2_ to H_2_

Although H_2_ and ethanol production were greatly improved in SH9*_ZG, the production of 0.15 mol mol^−1^ acetate and the lower-than-theoretical-maximum ethanol yield (1.67 mol mol^−1^) indicated that NAD(P)H production in SH9*_ZG was still insufficient. That theoretical maximum yield, for both H_2_ and ethanol, can be achieved when carbon flux is fully diverted to the PP pathway. Therefore, in order to study the effect of differential expression of Zwf and Gnd on NAD(P)H production and co-production of H_2_ and ethanol, SH9*_ZG was induced by varying isopropyl-β-D-thiogalactopyranoside (IPTG) from 0 to 0.2 mM (Fig. 3). As the inducer concentration increased, the rate of cell growth and glucose consumption decreased (Additional file 1: Figure S1). In the absence of inducer, the cell density reached 2.0 OD_600_ within 9 h, and the added glucose (28 mM) was completely consumed within 12 h. On the other hand, when the culture was induced with 0.2 mM IPTG, the cell density reached only 1.0 OD_600_ in 24 h, and furthermore, 8 mM glucose was left unconsumed at that time. As shown in Fig. 3a, ethanol production increased gradually, and acetate production correspondingly decreased, as the IPTG concentration was increased within the 0–0.05 mM range. These gradual changes in ethanol and acetate production reflected the gradual increase in NAD(P)H supply following the increase in carbon flux through the PP pathway. Also, it was noted that with the 0–0.05 mM IPTG concentration increase, the combined ethanol plus acetate production yields decreased while the ratio of CO_2_ to H_2_ gradually increased (Fig. 3b). This can be attributed to the loss of carbon in the form of CO_2_ in the oxidative PP pathway (see Fig. 1). It also supports the gradual shift of the carbon flux from the EMP to the PP pathway as the IPTG was increased. However, at >0.05 mM IPTG, no further changes in co-production profiles were observed; consequently, the acetate yield could not be reduced below 0.12 mol mol^−1^. This suggests that even at the highest IPTG concentration of 0.2 mM, the carbon flux was not fully diverted to the PP pathway. The consistency of the experimental data was examined by analyzing the carbon recovery and reduction degree balance (Additional file 2: Appendix). The carbon recovery was above 90 %, and the error in the reduction degree balance was within 5 %, indicating the reliability of the experimental data. Additionally, the flux distribution through the three glycolytic pathways was analyzed based on the metabolite profiles (Additional file 2: Appendix). In the case of SH9*_ZG, the maximum carbon flux through the PP pathway was estimated as ~70 % of total glucose metabolized.

**Fig. 4:**
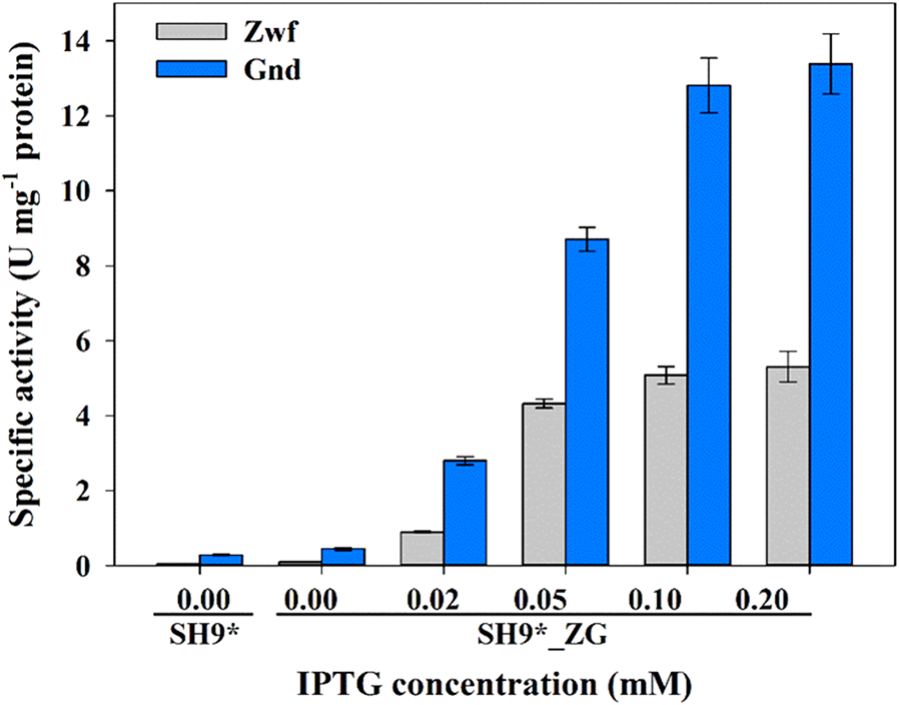
Specific activities of Zwf and Gnd in the cytosolic fractions of SH9* and SH9*ZG induced with varying concentrations of IPTG

One persistently puzzling question was why the co-production yields of H_2_ and ethanol in SH9*_ZG did not improve any more at >0.05 mM IPTG. We suspected that at IPTG above 0.05 mM, the expression and/or activities of Zwf and Gnd did not increase, and that this limited the improvement of the co-production yields. Therefore, the enzymatic activities of Zwf and Gnd were measured. The results showed that with increasing IPTG concentrations to 0.2 mM, the enzymatic activities of both Zwf and Gnd steadily increased (Fig. 4). The maximum activities of Zwf and Gnd in SH9*_ZG at 0.2 mM IPTG reached 5.3 and 13.4 U mg^−1^ protein, respectively. This suggests that the incomplete conversion of the carbon flux to the PP pathway was not caused by low Zwf and Gnd activities. This is somewhat discouraging, because complete conversion of the carbon flux to the PP pathway might not be possible simply by increasing the activities of Zwf and Gnd.

### Gene expression in SH9* overexpressing Zwf and Gnd

Transcription of key enzymes in the glycolytic and other pertinent pathways was examined after induction of Zwf and Gnd at different inducer concentrations (Table 3) (Refer to Fig. 1 for the enzymes examined). The deletion of *pfkA* in SH9*_ZG was confirmed by the lack of *pfkA* expression. As suggested from the genome sequencing results (see Fig. 2c), *pfkB*, the isozyme of *pfkA,* was highly expressed. In comparison, the *pfkB* expression was almost negligible when *pfkA* was intact in *E. coli* [20]. The *pfkB* expression level was reduced in SH9*_ZG with increasing IPTG concentration, suggesting that the EMP pathway might be down-regulated upon overexpression of Zwf and Gnd. It was also noted that the expression of *zwf*, *gnd*, *pgi*, *gapA*, and *adhE* increased when the inducer concentration increased. The increased expression of *pgi* and the decreased expression of *pfkB* suggest the active operation of the PP pathway in partial cyclic mode [25]. Phosphoglucose isomerase (Pgi) is a reversible enzyme and can perform the conversion of fructose-6-phosphate to glucose-6-phosphate when the partial cyclic PP pathway is functional. The increase in *gapA* expression is also related to upregulation of the PP pathway which is linked to the EMP pathway at the glyceraldehyde-3-phosphate (GAP) node. Upregulation of the PP pathway can increase GAP level and the expression of GapA so that glycolytic flux can be enhanced. The increased *adhE* expression is attributed to the increased intracellular NAD(P)H concentration following the high PP pathway flux. It has been reported that the expression of *adhE* increases when intracellular NAD(P)H level increases [26]. The enhanced ethanol production in SH9*_ZG is partly attributable to the improved *adhE* expression, but mostly by increased NAD(P)H supply (see below).

**Table 3:** Relative transcription levels of key glycolytic enzymes in SH9*_ZG after induction with different IPTG concentrations

Gene	IPTG concentration (mM)		
	0 mM	0.02 mM	0.20 mM
*pgi*	2.3	5.2	5.3
*pfkA*	0.00	0.00	0.00
*pfkB*	63.3	58.5	40.2
*gapA*	4.5	17.5	34.1
*edd*	0.30	0.6	0.5
*zwf*	1.02	2222.0	7631.1
*gnd*	8.08	1610.4	3075.0
*tktA*	9.4	6.1	4.3
*pflB*	28.4	21.2	21.1
*fhlA*	0.1	0.2	0.1
*udhA*	0.1	0.2	0.3
*adhE*	2.1	4.6	35.9
*pntA*	4.1	ND	1.6
*adhP*	0.2	ND	0.5
*eutG*	0.0	ND	0.1
*fucO*	0.1	ND	0.3
*yiaY*	0.0	ND	0.3
*yqhD*	0.6	ND	1.1
*yjgB*	0.3	ND	0.4
*mhpF*	0.1	ND	0.1
*aldB*	0.3	ND	0.4

The result was from three individual experiment repeats and the standard deviation was less than 10 %

*rpoD* was used as the endogenous control and the transcriptional level of rpoD was considered as 1

*ND* not determined

One important additional question is whether NADPH produced in the PP pathway is directly used for ethanol production or only after conversion to NADH. To answer this question, we determined the expression levels and activities of soluble transhydrogenase (UdhA; the enzyme known to convert NADPH to NADH) and membrane-bound transhydrogenase subunit (PntA), which converts NADH to NADPH [27]. As shown in Table 3, the transcription of *pntA* decreased as the inducer concentration increased; its activity, though not very high, could yet be detected. The stimulation of the PP pathway in SH9*_ZG should have provided sufficient NADPH, in which context, the decrease in *pntA* expression with increasing IPTG is understandable. On the other hand, the UdhA mRNA levels in SH9*_ZG, albeit increasing gradually with increasing IPTG concentration, were very low, and furthermore, no enzymatic activity was detectable in any of the SH9*_ZG, including the one induced with the highest IPTG concentration. Similar result has been reported in other *E. coli* strain [28]. If *E. coli* alcohol dehydrogenase (AdhE) uses NADH as a cofactor and the conversion of NADPH to NADH is necessary, UdhA activity should be highly elevated in SH9*_ZG. To elucidate the role of UdhA then, we overexpressed *udhA* from a multi-copy plasmid in SH9*_ZG (SH9*_ZGU). However, the recombinant overexpressing UdhA did not exhibit any change in ethanol production (Additional file 3: Figure S2). From this result, we hypothesized that NADPH is directly utilized for ethanol production by AdhE and/or unknown NADPH-dependent alcohol dehydrogenase (ADH). According to literature survey [29, 30], there exist several putative candidate ADHs including *adhP*, *eutG*, *fucO*, *yiaY*, *yqhD*, *yjgB*, *mhpF*, and *aldB.* The expression levels of these putative ADHs were analyzed, but none of them showed improved mRNA levels when induced with high IPTG concentrations (Table 3). At this moment, it remains unclear whether the major ethanol-producing enzyme in the current *E. coli* is NADPH- or NADH-dependent.

### Necessity of down-regulation of EMP pathway by *pfkA* deletion for enhanced PP pathway

The results from genome sequencing and gene expression analysis confirmed that PfkB, the minor isozyme of PfkA, was actively expressed and facilitated the growth of both *pfkA*-deletion mutants SH8* and SH9*. A question, then, is whether the EMP pathway in SH9* is actually down-regulated or not, and, if so, whether it is necessary or if simple overexpression of Zwf and Gnd is enough to improve the carbon flux through the PP pathway. To answer these questions, we overexpressed Zwf and Gnd in the SH5 strain wherein *pfkA* was not disrupted. This, SH5_ZG, clearly demonstrated the importance of overexpression of Zwf and Gnd: ethanol production was improved to 1.09 mol mol^−1^ (from 0.79 mol mol^−1^ of SH5), while acetate production was decreased to 0.35 mol mol^−1^ (from 0.67 mol mol^−1^ of SH5) (Table 2). However, in comparison with the SH9*_ZG strain, SH5_ZG’s co-production of H_2_ and ethanol was much lower, even though both strains had similar activities of Zwf and Gnd (Additional file 4: Figure S3). These results indicate the following: (1) overexpression of Zwf and Gnd can activate the PP pathway regardless of down-regulation of the EMP pathway, and (2) the EMP pathway must be down-regulated to enhance the glycolytic flux through the PP pathway.

### Disruption of ED pathway for increased flux through PP pathway

From the results obtained and discussed thus far, it is clear that to achieve a higher co-production yield of H_2_ and ethanol, additional carbon flux needs to be diverted to the PP pathway by further down-regulation or even the complete blockage of the EMP and/or ED pathway. Because complete blockage of the EMP pathway by *pgi* deletion prevents anaerobic growth [13], we disrupted the ED pathway by deleting *edd* and *eda* in SH9* and overexpressed Zwf and Gnd (SH10_ZG). The SH10_ZG could grow under anaerobic conditions, though its growth rate was slower than that of SH9*_ZG (Additional file 5: Figure S4). Furthermore, contrary to our expectation, co-production of H_2_ and ethanol decreased while that of acetate increased (Table 2). This behavior of SH10_ZG suggests the reduction of NAD(P)H availability by ED pathway disruption. When the ED pathway is blocked, the additional carbon should be diverted through the EMP instead of the PP pathway. Deletion of the ED pathway can accumulate 6-phosphogluconate, which pushes glucose more through EMP pathway at the glucose-6-phosphate node.

## Conclusion

In this study, we demonstrated successful co-production of H_2_ and ethanol by eliminating pyruvate accumulation in the *E. coli* strain SH9*_ZG. This was possible via down-regulation of the EMP pathway by deletion of *pfkA* and overexpression of the two major PP pathway enzymes, Zwf and Gnd, while maintaining the acetate production pathway intact. The maximum yields of H_2_ and ethanol, 1.88 and 1.40 mol mol^−1^, respectively (both close to the theoretical maximum of 1.67 mol mol^−1^ for each), were obtained by SH9*_ZG. Analysis of the carbon distribution and gene expression confirmed that the PP pathway was actively functioning in SH9*_ZG. However, due to insufficient NAD(P)H supply, some acetate, up to 0.12 mol mol^−1^, was produced. To further improve co-production yields, still-unknown hurdles to the operation of the PP pathway as the sole glycolytic route should be identified and removed.

## Methods

### Strains, plasmids, and materials

The mutant strains were developed from the base strain, SH5, constructed in our previous study [31]. The restriction enzymes, Phusion^®^ high-fidelity DNA polymerase and other DNA-modifying enzymes used for gene cloning, were obtained from New England Bio-Labs (Beverly, MA, USA). The pDK7 plasmid was obtained from Kleiner et al. [32]. The genome for the PCR template was isolated using a genomic DNA isolation kit purchased from Promega (Madison, WI, USA). Mini-preparation of plasmids was performed using the LaboPass™ plasmid extraction kit (Cosmo genetech Co. Ltd., Korea). The oligonucleotides for PCR were synthesized, and amplified PCR fragments were sequenced by Macrogen Inc. (Seoul, Korea). Yeast extract (Cat. 212750) and Bacto™ tryptone (Cat. 211705) were purchased from Difco (Becton–Dickinson; Franklin Lakes, NJ, USA). Unless indicated otherwise, all of the other chemicals were acquired from Sigma (St. Louis, MO, USA).

### Construction of recombinant *E. coli* strains

Plasmids pEcZ, pEcG, and pEcZG from our previous study were used for homologous overexpression of Zwf and Gnd [18]. The recombinant plasmids were constructed from pDK7 with the IPTG-inducible *tac* promoter. The deletions of *pfkA* and *edd eda* were performed using λ-Red recombinase and pKOV methods, respectively. pKD46 vector and linear DNA fragment with homologous regions as well as the recombinant pKOV plasmid were constructed to delete the target gene using the previously described methods [33, 34]. The list of strains constructed in this study is provided in Table 1.

### Culture conditions

LB medium was used for culturing cells during recombinant strain construction, and M9 medium was used in co-production experiments. The M9 medium composition is 5.0 g L^−1^ glucose, 1.0 g L^−1^ yeast extract, 3.0 g L^−1^ Na_2_HPO_4_, 1.5 g L^−1^ KH_2_PO_4_, 0.5 g L^−1^ NH_4_Cl, 0.25 g L^−1^ NaCl, 0.25 g L^−1^ MgSO_4_, 0.01 g L^−1^ CaCl_2_, 0.2 mg L^−1^ NiSO_4_, 1.4 mg L^−1^ FeSO_4_, 0.2 mg L^−1^ Na_2_SeO_3_, 0.2 mg L^−1^ Na_2_MoO_4_, and 8.8 mg L^−1^ cysteine HCl. Cells were cultured at 37 °C in either a 250 mL Erlenmeyer flask or a 165-mL serum bottle in an orbital shaker rotating at 200 rpm. The recombinant cells were maintained with kanamycin (50 µg mL^−1^) and chloramphenicol (25 µg mL^−1^) whenever cultured. For anaerobic experiments, the serum bottles containing M9 media were purged with argon for 15 min to remove oxygen present in the headspace and media. The expression levels of Zwf and Gnd were initiated by the addition of 0.1 mM IPTG at the beginning of cultivation. In the differential expression experiment the IPTG concentration was varied at 0–0.2 mM.

### RNA extraction and real-time PCR

Samples for real-time PCR (RT-PCR) analysis were collected in the late exponential growth phase during the fermentation experiment. The RNA in the samples was stabilized by adding two volumes of RNAprotect reagent (Qiagen Korea Ltd., Korea) and processed as dictated in the protocol. The cell pellets, as treated with RNAprotect, were stored at −80 °C prior to extraction of total RNA. A Nucleospin^®^ RNA isolation kit (MachereyNagel, Germany) was used to isolate the total RNA from the processed pellets. The RNA was quantified in a UV-spectrophotometer and checked in agarose gel for integrity and concentration. The isolated total RNA and random hexamers were used to synthesize cDNA using the SuperScript III first-strand synthesis system (Thermo Fisher Scientific, Waltham, MA, USA). RT-PCR analysis was performed using the synthesized cDNA, gene-specific primers and Power SyBr^®^ Green (Thermo Fisher Scientific). RT-PCR was performed in a 48-well StepOne real-time PCR system (Thermo Fisher Scientific), and *rpoD* was used as an endogenous control. The experiment was performed in duplicate, and relative mRNA quantification was performed according to the Δ*C*_T_ method [35].

### Genome sequencing of adapted strains

The genomes of the SH9 and SH9* strains were sequenced by next-generation sequencing [36]. The genome isolation and sequencing were performed by Macrogen Inc., Korea, using the Illumina HiSeq 2000 sequencer. The genome sequence of *E. coli* BW25113 (GenBank accession no. **CP009273.1**) was used as seen in Ref. [37]. The SH9 and SH9* genomes were compared, and the single-nucleotide polymorphisms (SNPs) and insertions/deletions (INDELs) were analyzed in the adapted strain.

### Determination of enzymatic activities

The enzymatic activities of Zwf and Gnd were measured as outlined previously, though with slight modifications to the protocol [38, 39]. Briefly, Zwf activity was determined with 0.5 mM of glucose-6-phosphate as the substrate and 0.2 mM of NADP^+^ as the cofactor. Gnd activity was determined in the same manner, except with 6-phosphogluconate as the substrate. The assay buffer contained 50 mM Tris–HCl buffer (pH 7.5) and 10 mM MgCl_2_. Soluble fractions of the culture were incubated at 30 °C for 2 min in the reaction buffer, and the assay was initiated by the addition of the substrate and co-factor. The reduction of NADP^+^ was monitored at 340 nm, and the enzymatic activity was calculated. One unit of enzymatic activity is defined as the number of µmoles of NADP^+^ reduced per minute. Soluble transhydrogenase (UdhA) activity was measured according to the protocol defined by Boonstra et al. [28]. Briefly, the reduction of thio-NAD^+^ by UdhA was measured at 400 nm with NADPH as the substrate.

### Analytical methods

The cell growth was determined periodically by measurement of the optical density (OD_600_) of the cultures at 600 nm using a UV spectrophotometer (Lambda 20, Perkin Elmer, USA). The gases present in the headspace of the serum bottles were measured by gas chromatography (DS6200 Donam Systems Inc., Seoul, Korea) fitted with a column and thermal conductivity detector. The amounts of glucose, ethanol, and other metabolites were quantified using high-performance liquid chromatography (Agilent Technologies, HP, 1200 series) installed with an Aminex carbohydrate analysis column, as described in Sankaranarayanan et al. [40]. Protein-expression analysis was performed by SDS-PAGE as described earlier [41]. The protein present in the samples used for determination of enzymatic activity was measured by Bradford assay as described previously [42].

## Additional files

10.1186/s13068-016-0510-5 Cell growth (A) and glucose consumption (B) of SH9*_ZG induced with different concentrations of IPTG.
10.1186/s13068-016-0510-5 Consistency of experimental data and estimation of carbon flux through PP pathway using ethanol yield.
10.1186/s13068-016-0510-5 Ethanol production by SH9*_ZG and SH9*_ZGU induced with 0.2 mM IPTG.
10.1186/s13068-016-0510-5 Specific activities of Zwf and Gnd in the cytosolic fractions of SH9*_ZG and SH5_ZG.
10.1186/s13068-016-0510-5 Cell growth of SH9*_ZG and SH10_ZG under anaerobic conditions.

## Abbreviations

ADH: alcohol dehydrogenase
CO_2_: carbon dioxide
ED: Entner–Doudoroff
EMP: Embden–Meyerhof–Parnas
GAP: glyceraldehyde-3-phosphate
H_2_: hydrogen
INDEL: insertion/deletion
IPTG: isopropyl-β-D-thiogalactopyranoside
OD_600_: optical density
Pgi: phosphoglucose isomerase
PP: pentose-phosphate
RT-PCR: real-time PCR
SNP: single-nucleotide polymorphism
