# Supplementary material for: Co-production of hydrogen and ethanol by pfkA-deficient Escherichia coli with activated pentose-phosphate pathway: reduction of pyruvate accumulation
Source: Biotechnol Biofuels. 2016 Apr 29;9:95. doi: 10.1186/s13068-016-0510-5 (PMC4850720; doi:10.1186/s13068-016-0510-5)
Supplement: Supplementary file 2 — 10.1186/s13068-016-0510-5 Consistency of experimental data and estimation of carbon flux through PP pathway using ethanol yield. [file 13068_2016_510_MOESM2_ESM.docx]

**Appendix. Consistency of experimental data and estimation of carbon flux through PP pathway using ethanol yield**

Data consistency is essential for accurate quantitative analysis of culture experiments. All culture results obtained in this study were analyzed for data consistency and the one from SH9*_ZG was given as an example (Table A1). The experimental data presented in this study showed a good consistency analyzed by carbon recovery and reduction degree balance. The decrease in carbon recovery with increasing IPTG concentration is attributed to increasing amount of dissolved CO_2_ in the culture medium, which was not considered in the calculation of carbon recovery.

Table A1: Carbon distribution and reduction degree balance in SH9*_ZG induced under different IPTG concentration^a^

| **Yield (mol/mol)** | **IPTG concentration (mM)** | | | |
| --- | --- | --- | --- | --- |
|  | **0.00** | **0.02** | **0.10** | **0.20** |
| H_2_ | 1.84 | 1.91 | 1.84 | 1.73 |
| Ethanol | 1.02 | 1.13 | 1.39 | 1.38 |
| Acetate | 0.87 | 0.65 | 0.15 | 0.12 |
| Pyruvate | 0 | 0 | 0 | 0 |
| CO_2_^b^ | 1.54 | 1.70 | 1.82 | 1.73 |
| **Carbon distribution (%)** | | | | |
| Pyruvate | 0 | 0 | 0 | 0 |
| Ethanol | 33.93 | 37.77 | 46.60 | 46.00 |
| Acetate | 28.90 | 21.60 | 4.87 | 4.03 |
| Biomass^c^ | 9.65 | 8.32 | 7.23 | 7.40 |
| CO_2_^b^ | 25.77 | 28.26 | 30.33 | 28.78 |
| *Recovery (%)* | 98.25 | 95.95 | 89.03 | 86.22 |
| **Reduction degree balance** | | | | |
| **Reactant** |  |  |  |  |
| Glucose | 24.00 | 24.00 | 24.00 | 24.00 |
| **Product** |  |  |  |  |
| Pyruvate | - | - | - | - |
| Ethanol | 12.22 | 13.60 | 16.78 | 16.56 |
| Acetate | 6.94 | 5.18 | 1.17 | 0.97 |
| Cell mass | 2.36 | 2.03 | 1.77 | 1.82 |
| H_2_ | 3.69 | 3.81 | 3.67 | 3.45 |
| CO_2_ | 0 | 0 | 0 | 0 |
| Total | 25.21 | 24.62 | 23.39 | 22.80 |
| *Errors (%)* | 4.99 | 2.60 | -2.58 | -5.01 |

^a^ The values given here are the average of three independent culture experiments. Standard deviation of each value was less than 10%

^b^ CO_2_ in the head space only; soluble CO_2_ in the culture medium was not included.

^c^ Cell composition was assumed as CH_1.77_O_0.49_N_0.24_P_0.017_. The average ash content of 5.5% was deduced from the actual dry mass [1].

Carbon flux distribution in different glycolytic pathways can be directly and accurately determined if ^13^C tracer experimentation is performed [2]. However, in this study, due to the lack of ^13^C tracer experiment capability, indirect estimation of carbon flux distribution was attempted based on the production yield of ethanol and NAD(P)H.

According to the ^13^C tracer experiments by Chen *et al.*, anaerobically-growing *E. coli* cells metabolize most of glucose through EMP pathway [2]. Our experiments with SH5 and subsequent analysis of flux distribution by MetaFluxNet also confirmed the predominant role of EMP pathway [3]. Therefore, it was assumed that other host strains such as SH8* and SH9* metabolized glucose through EMP pathway only. It should be noted that some yeast extract, 1.0 g L^-1^, was supplemented to our culture medium as additional nitrogen source along with NH_4_Cl (1.0 g L^-1^), and cell growth was very low as 0.030 ~ 0.037 g L^-1^ under anaerobic conditions. This suggests that the role of PP pathway to provide C5-sugars and NADPH for cell growth might not be high. If glucose is metabolized to pyruvate exclusively by the EMP (or ED) pathway, 2 mol of NAD(P)H are produced from 1 mol of glucose. This means that 1 mol of ethanol should be produced from 1 mol of glucose. In the present results, SH9* produced 0.85 mol of ethanol from 1 mol of glucose (which requires 1.7 mol NAD(P)H), indicating that about 15% of the NAD(P)H produced was used for biomass production and/or maintenance of cell viability. When sorbitol (which produces 3 mol of NAD(P)H during its conversion to pyruvate) was used as the carbon source, 1.33 mol of ethanol was produced by SH9*. This indicates that about 12% of the NAD(P)H produced was used for biomass production and/or maintenance of cell viability. In the same way, with SH5, where 0.79 mol of ethanol was produced from 1 mol of glucose, 21% of the NAD(P)H was assumed to have been utilized for biomass production or maintenance. With SH8*, 11% of NAD(P)H was assumed to have been used for biomass production or maintenance. Overall, it seems reasonable to assume that, for anaerobically-growing *E. coli* cells, the fraction of NAD(P)H used for cell growth and maintenance is somewhere within the 10 – 20% range (see Table A2).

Table A2: Calculation of amount of NAD(P)H generated in SH9*_ZG

| **Strains** | **Carbon source** | **Yield**  (mol mol^-1^ glucose or sorbitol) | | **Fraction of NAD(P)H used for ethanol production** |
| --- | --- | --- | --- | --- |
|  |  | **Ethanol** ^a^ | **NAD(P)H** |  |
| SH5 | Glucose | 0.79 | 2^b^ | 0.79 |
| SH8* | Glucose | 0.89 | 2^b^ | 0.89 |
| SH9* | Glucose | 0.85 | 2^b^ | 0.85 |
| SH9* | Sorbitol | 1.33 | 3^b^ | 0.88 |
| SH9*_ZG | Glucose | 1.40 | 3.22^d^ | 0.85^c^ |

^a^ Ethanol yield obtained from experimental data

^b^ Theoretical yield of NAD(P)H assuming that EMP (or ED) pathway is the sole glycolytic route.

^c^ Assumed based on the values for SH5, SH8* and SH9*.

^d^ Estimated from the assumption that the fraction of NAD(P)H used for ethanol production is 0.85.

It is natural to assume that the amount of NAD(P)H used for cell growth and maintenance increases as cell growth increases. The amount of NAD(P)H predicted to be used for growth and maintenance was plotted against biomass production for SH5, SH8* and SH9* (Fig. A1). NAD(P)H used for growth and maintenance was determined by subtracting the NAD(P)H usage for ethanol production from the total NAD(P)H production (see Table A2). Although showing increasing trend, the amount of NAD(P)H used for growth and maintenance was not proportional to biomass production. With the current *E. coli* strains growing under anaerobic conditions, the ratio of cell mass to NAD(P)H or Y_cell/NAD(P)H_ (g mmol^-1^) was estimated to be 0.063 ~ 0.10.





Figure A1: NAD(P)H used for biomass production vs. cell growth for SH5, SH8* and SH9*.

To estimate NAD(P)H yield in SH9*_ZG where a large fraction of glucose is metabolized via PP pathway, we assumed that 15% of NAD(P)H generated were used for cell growth and maintenance. In addition, it was assumed that PP pathway was operated in non-cyclic mode only (i.e., the reverse reaction of phosphoglucoisomerase converting fructose-6-phosphate to glucose-6-phosphate was ignored). The non-cyclic PP pathway can generate 3.67 mol mol^-1^ NAD(P)H from one mol of glucose [4]. If *α* is the fraction of glycolytic flux through the PP pathway, the yield of NAD(P)H (mol mol^-1^ glucose) can be expressed as

Yield of NAD(P)H $=\left[ \left( \alpha\right)NAD\left( P \right)H yield of PP \right]+\left[ (1-\alpha)NAD\left( P \right)H yield of EMP/ED \right]$

Because the yield of NAD(P)H in SH9*_ZG is 3.22, *α* was estimated as 0.73. This means that, in SH9*_ZG, a maximum of 70% glucose is metabolized through the PP pathway while the remaining 30% is through the EMP or ED pathway.

**Reference:**

1. Stephanopoulos G, Aristidou A, Nielsen J: *Metabolic Engineering*. San Diego: Academic Press; 1998. P. 120
2. Chen, X., Alonso, A. P., Allen, D. K., Reed, J. L., & Shachar-Hill, Y., Synergy between ^13^C-metabolic flux analysis and flux balance analysis for understanding metabolic adaption to anaerobiosis in *E. coli*. *Metabolic engineering* 2011, *13*, 38-48.
3. Seol, E., Ainala, S. K., Sekar, B. S., Park, S., Metabolic engineering of *Escherichia coli* strains for co-production of hydrogen and ethanol from glucose. *International Journal of Hydrogen Energy* 2014, *39*, 19323-19330.
4. Stryer L: *Biochemistry*. New York: W.H. Freeman; 1995. P. 565
